# Prescription Department

**Published:** 1891-07

**Authors:** 


SUMMER DIARRHOEA—

℞ Carbolic acid, gtts. 2–4.
Tr. opii div'd dr. i–iss.
Bis. subnit., dr. ii–oz. ss.
Mucilage accacis oz. i.
Aqua menth vir, q. s oz. ii.

Mix. Sig.: Teaspoonful every 3 or 4 hours.

T. D. LONGINO, M. D.

℞ Bismuth subnit., grs. xxiv.

Pure pepsin, grs. xij.

Salol, grs. vj.

M. Ft. 12 powders. Sig.: one every 2 to 4 hours for a child one year old, suffering from simple diarrhœa.

N. O. HARRIS, M. D.

℞ Acid mur. dil. f. dr. i.
Fairchild's ess. of pepsin, f. oz. i.
Syr. of orange peel, q. s. f. oz. iv.

Mix and give a teaspoonful with a little water every 2 or 3 hours to child from one to 2 years old.

J. McF. GASTON, M. D.

℞ Salolis, grs. xxxvi,
Hydg. chlo. nit., grs. ii.
Bis. subnit. dr. ii.
Fairchild's essence pepsin.
Syr. zinziboris q. s. aa. oz. iii.

M. Sig.: Shake and take a teaspoonful every two to four hours.

J. S. TODD, M. D.

The following is recommended:
℞ Resorcin., gr. iss–iij.
Infus. chamomil., f. oz. ij.
Tr. opii, gtt. ij.
Tr. cascarill., gtt. xv.

M. Sig.: Teaspoonful every two hours.—*Kinder Arzt*, April, 1890.

Critzmann employs the following:
℞ Bismuth salicylat., gr. xv–dr. ss.
Tinct. opii, gtt. i–v.
Infus. theæ, f oz. ij.
Syrup rubi, f dr. v.
Rum, f dr. iv–v. M.

—*Jour. Am. Med. Ass'n.* March 23, '91.

FISSURED NIPPLES—

℞ Balsam. Peru.

Tinct. arnicæ, aa dr. ss.

Ol. amygdal. dulc. oz. j.

Aquæ calcis, oz. ss. M.

Sig.: Apply a small quantity several times daily.—*Ex.*

For Erysipelas—

R Ichthyol, dr. ij.
Ether, dr. ij.
Collodion, dr. iv. M.

S. Paint over and around infected area.—*Prov. Med. Journal.*

For Amenorrhoea—

A writer in *Revue Med.-chir. des Mal. des Femmes* (in *Med. News*, May 16th), prescribes—

R Bichloride of mercury, 3 grains.

Arsenite of sodium, 3 grains.

Sulphate of strychnine, 1½ grains.

Carbonate of potass. } each 45 grs.

Sulphate of iron, }

Make into 60 pills and give one pill after each meal.—*Ex.*

Dental Caries—

The following prescription has been suggested:

R Acid. tannic., gr. lxxv.
Tinct. iodinii.
Tinct. myrrhæ, aa f dr. j.
Potassii iodidi, gr. xv.
Aquæ rosæ, f oz. vj. M.

Add a teaspoonful to a small glass of water and use daily as a mouth wash. —*Pharm. Record*, March 5, '91.

Fumigant for Asthma—Plant:

R Stramonium leaves, } of each 1 oz.

Green tea, }

Lobelia inflata, 3 drachms.

Add a saturated solution of potassium nitrate, dry, and preserve in a well-stopped bottle. A teaspoonful suffices for a fumigation.—*La Sem. Med.*, April 29, 1891.

A favorite prescription of the outdoor medical department of the Jefferson Medical College Hospital, in cases of difficult digestion accompanied by constipation and flatulency, is the following:

R Tinct. nucis vomicæ,
Tinct. belladonna.
Tinct. physostigmatis, aa f dr. ij.
Ext. cascaræ fluid, f dr. vj.
Ext. erythroxylon, q. s. f oz. iij.

M. Sig.: A teaspoonful t. d. after meals.

